# LINC Complexes Mediate the Positioning of Cone Photoreceptor Nuclei in Mouse Retina

**DOI:** 10.1371/journal.pone.0047180

**Published:** 2012-10-05

**Authors:** David Razafsky, Nathaniel Blecher, Alexander Markov, P. J. Stewart-Hutchinson, Didier Hodzic

**Affiliations:** 1 Department of Ophthalmology and Visual Sciences, Washington University School of Medicine, St Louis, Missouri, United States of America; 2 Department of Pathology, New York University School of Medicine, New York, New York, United States of America; Baylor University, United States of America

## Abstract

It has long been observed that many neuronal types position their nuclei within restricted cytoplasmic boundaries. A striking example is the apical localization of cone photoreceptors nuclei at the outer edge of the outer nuclear layer of mammalian retinas. Yet, little is known about how such nuclear spatial confinement is achieved and further maintained. Linkers of the Nucleoskeleton to the Cytoskeleton (LINC complexes) consist of evolutionary-conserved macromolecular assemblies that span the nuclear envelope to connect the nucleus with the peripheral cytoskeleton. Here, we applied a new transgenic strategy to disrupt LINC complexes either in cones or rods. In adult cones, we observed a drastic nuclear mislocalization on the basal side of the ONL that affected cone terminals overall architecture. We further provide evidence that this phenotype may stem from the inability of cone precursor nuclei to migrate towards the apical side of the outer nuclear layer during early postnatal retinal development. By contrast, disruption of LINC complexes within rod photoreceptors, whose nuclei are scattered across the outer nuclear layer, had no effect on the positioning of their nuclei thereby emphasizing differential requirements for LINC complexes by different neuronal types. We further show that Sun1, a component of LINC complexes, but not A-type lamins, which interact with LINC complexes at the nuclear envelope, participate in cone nuclei positioning. This study provides key mechanistic aspects underlying the well-known spatial confinement of cone nuclei as well as a new mouse model to evaluate the pathological relevance of nuclear mispositioning.

## Introduction

Many CNS tissues display a laminar organization that consists in various number of nuclear layers separated by synaptic zones. A good example is the mammalian retina – an accessible and well-defined part of the CNS – that is composed of three distinct nuclear layers separated by two zones of synaptic contacts, the inner and outer plexiform layers (IPL and OPL, respectively). Six neuronal types populate the retina: cone and rod photoreceptors whose nuclei form the outer nuclear layer (ONL), horizontal, bipolar and amacrine cells whose nuclei form the inner nuclear layer (INL), and retinal ganglion cells (RGC) whose nuclei delineate the ganglion cell layer (GCL). Müller cells, that form the retinal glia, position their nuclei within the INL. Cone photoreceptors provide spectacular examples of polarized nuclear positioning. Indeed, their nuclei invariably localize on the apical side of the ONL while their axons extend across the thickness of the ONL to establish synaptic contact with second order neurons within the OPL [1], [2]. One can wonder whether this specific nuclear positioning has any functional relevance since, by comparison, rod photoreceptors do not require any particular spatial confinement of their nuclei to function. Answering this question first requires the identification of molecular mechanisms underlying the establishment and maintenance of nuclear spatial confinement.

**Figure 1:**
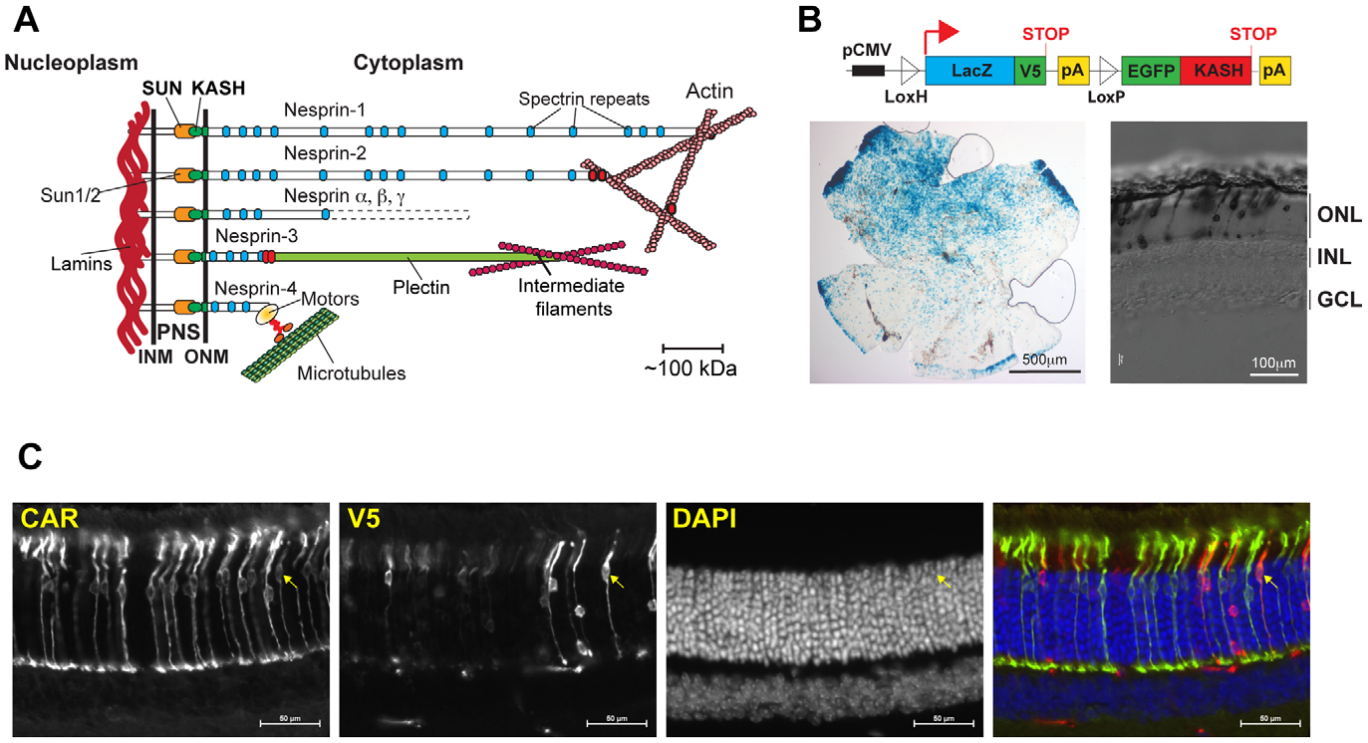
Transgenic expression pattern of Tg(CMV-LacZ/EGFP-KASH2) retinas. A) Depiction of the organization of LINC complexes that physically couple the nuclear lamina to peripheral cytoskeletal networks and molecular motors. INM, ONM: Inner and outer nuclear membrane, respectively. PNS: perinuclear space. Nesprin α, β and γ depict shorter isoforms originating from the alternative splicing of Nesprin 1 and 2 genes. B) Top: depiction of the CMV-LacZ/EGFP-KASH2 genetic construct (see text for details). Left panel: Transgenic expression pattern detected by X-gal staining of Tg(CMV-LacZ/EGFP-KASH2) retinal flat mount. Note the preferential transgenic expression on the dorsal side of the retina. Right panel: X-gal staining of vertical slices. Note the restriction of transgenic expression to the outer nuclear layer. ONL: outer nuclear layer; INL: inner nuclear layer; GCL: ganglion cell layer. C) LacZ/V5 is mostly expressed in rods and a few cones. Vertical sections of P32 Tg(CMV-LacZ/EGFP-KASH2) were immunostained with anti-Cone arrestin (CAR) and anti-V5 antibodies. The arrow points to a CAR+/V5+ transgenic cone. Scale bars: 50 μm.

Recently, major progress has been achieved in the identification of nuclear envelope (NE) proteins that mediate nuclear migration and/or anchorage (Fig. 1A). The NE is composed of the inner and outer nuclear membranes (INM and ONM, respectively) that merge at nuclear pores and delineate the perinuclear space. The ONM is an extension of the rough ER and the INM tightly adheres to the nuclear lamina, a meshwork of nuclear type-V intermediate filaments represented by A- and B-type lamins [3], [4]. Linkers of the Nucleoskeleton to the Cytoskeleton (LINC complexes) refer to macromolecular assemblies that span the nuclear envelope and physically connect the nuclear lamina to cytoplasmic cytoskeletal networks and molecular motors [5]–[7]. They form through direct interactions between two families of mammalian proteins: Sun proteins and Nesprins. Sun1 and Sun2 are integral transmembrane proteins of the inner nuclear membrane (INM) whose nucleoplasmic regions interact directly with components of the nuclear lamina [8]–[10]. On the other side of the INM, within the perinuclear space, Sun proteins interact directly with Nesprins, a family of transmembrane proteins that populate the outer nuclear membrane [11]–[13]. These interactions occur through evolutionary conserved SUN (Sad1/Unc84) and KASH (Klarsicht/Anc-1/Syne Homology) domains that characterize Sun proteins and Nesprins, respectively [10], [14], [15]. In turn, the cytoplasmic region of Nesprins, whose sizes vary from ∼50 kDa to 1MDa, interact with different cytoskeletal networks and motor proteins [12], [13], [16]–[18]. SUN/KASH interactions have been functionally identified in invertebrates, vertebrates and most recently in plants [19]. Recent crystallographic analyses have demonstrated that SUN domains form trimeric structures that interact directly with KASH domains [20].

Genetic alterations of either SUN or KASH domain-containing orthologs in *C.elegans* and *D.melanogaster* have established the central role that SUN/KASH interactions play in nuclear positioning during development [5]–[7], [21]. In mice, nuclear positioning defects have been demonstrated within skeletal muscles of Nesprin1 KO mice and in cone photoreceptors of Sun1 and Nesprin2 KO mice [22]–[24]. Sun1/2 or Nesprin1/2 double knockout (DKO) mice display severe cortical lamination defects and major developmental abnormalities of the CNS resulting in perinatal lethality [25]. This phenotype originates from the critical role that LINC complexes play in the physical coupling between the centrosome and the nucleus within newborn cortical neurons [25], [26]. By contrast, single KO mice of either Sun proteins or Nesprins do not show any apparent developmental defects thereby emphasizing the redundant function of multiple Sun and Nesprin encoding genes during mammalian CNS development. To overcome these limitations and bypass the potential contribution of cell nonautonomous phenotypes associated to KO approaches, we developed a new mouse model allowing for the spatiotemporal disruption of LINC complexes and applied this transgenic strategy to examine the mechanisms of nuclear positioning within mouse photoreceptor cells. We show that, by contrast to rod photoreceptors, the positioning of cone photoreceptor nuclei relies on intact LINC complexes. We provide evidence that this phenotype originates from the inability of cone precursors nuclei to migrate towards the apical edge of the ONL during early postnatal retinal development and further identified ultrastructural defects of cone synaptic terminals resulting from cone nuclei mispositioning within the OPL. Finally, our results suggest a model whereby Sun1 acts redundantly with Sun2 in mediating cone nuclear positioning whereas A-type lamins are dispensable for the positioning of cone nuclei on the outer edge of the ONL.

## Materials and Methods

### Ethics statement

Animal protocols used in this study strictly adhered to the ethical and sensitive care and use of animals in research and were approved by the Washington University School of Medicine Animal Studies Committee (Animal Welfare Assurance Permit # A-3381-01, protocol# 20110163).

### Transgenic and knockout mice

The KASH domain of mouse Nesprin2 (amino acids 6810 to 6874 from NP_001005510.2) was amplified by RT-PCR from C2C12 mouse myoblasts and cloned into BglII/BamH1 sites of pEGFP-C1. The EGFP-KASH2 open reading frame was subcloned in HindIII and ApaI sites of the pCMV-flox polylinker [27]. A PacI fragment encompassing the whole genetic construct described in Fig. 1B was used for pronuclear injection (Mouse Genetics Core, Washington University School of Medicine). LMNA^+/−^ (B6.129S1(Cg)-*LMNA^tm1Stw^*/BkknJ, #009125) and Sun1^+/−^ (B6;129S6-*Sun1^tm1Mhan^*/J, #012715) were purchased from The Jackson Laboratory [25], [28]. Tg(HRGP-cre)#Yzl and Tg(rx3-cre)1Mjam were kind gift from Drs. Y. Le and M. Jamrich, respectively [29], [30]. Mouse colonies were maintained and genotyped with appropriate primer at the Mouse Genetics Core.

### Preparation of mouse retinas

Mice were sacrificed via CO_2_ inhalation and ocular globes were immediately isolated and rinsed in PBS. Several incisions were performed in the cornea before incubating the whole eye in 4% paraformaldehyde (PFA)/PBS for 1 h at 4°C. To analyze vertical retina slices, whole eyes were rinsed in PBS, incubated overnight in a 30% sucrose/PBS solution and embedded in OCT compound (Tissue-TEK). For immunofluorescence microscopy, cryosections (10 μm) on Superfrost Plus slides (VWR) were fixed for 10 min in 4% PFA in PBS, rinsed three times in PBS, permeabilized in 0.5% Triton-X100/PBS and incubated with primary antibodies diluted in 10% donkey serum/0.5% Triton X-100 in PBS. Secondary antibodies conjugated to Alexa594 or 488 (Invitrogen) were incubated in the same conditions. Following DAPI staining, slices were mounted in fluorescent mounting medium (DAKO). For retinal flat mounts, cornea, lens and vitreous were removed after PFA fixation. The retina was then separated from the sclera, washed three times for 30 min in TBST and permeabilized in 3% Triton X-100 in PBS overnight at 4C and incubated with primary antibodies diluted in 3% bovine serum albumin/TBST overnight. After three washes in TBST, secondary antibodies were applied overnight. Retinas were then washed and counterstained with DAPI before being mounted between two coverslips. For X-gal staining, fixed retinas were washed twice in PBS and incubated in three changes of wash solution (0.1% Triton X-100, 2 mM MgCl_2_, 0.1 M phosphate buffer pH 7.2). X-gal staining was performed overnight in wash solution containing 6 mM K_3_Fe(CN)_6_, 3 mM K_4_Fe(CN)_6_ and 1 mg/ml Xgal. All images were acquired on an Eclipse Ti (Nikon) inverted fluorescence microscope using either dry 20X (Plan Apo, N.A. 0.75) or oil 40X (Plan Fluor, N.A. 1.3) objectives.

### Antibodies

Anti-V5 and anti-Lamin B2 (Invitrogen), anti-Lamin A/C (Santa Cruz Biotechnology and Cell Signaling), anti-Lamin B1, anti-Gαt1 and anti-BOP (Santa Cruz Biotechnology), anti-cone arrestin (Millipore) and anti-Ribeye (BD Transduction Laboratories) were used in this study. Rabbit anti-mouse Sun1 was raised against a luminal epitope located downstream from the transmembrane domain. Rabbit anti-Nesprin1 and anti-Nesprin2 sera were raised against fusion proteins corresponding to epitopes located just upstream from their respective KASH domains and the Nesprin3 antiserum was raised against a fusion protein corresponding to the whole cytoplasmic region of mouse Nesprin3 (Primm Biotech). Sun1, Nesprin2 and Nesprin3 antisera were immunoaffinity purified prior to use. An anti mouse Sun2 serum was kindly provided by Dr. Min Han [31]. Alexa594 conjugated peanut agglutinin (PNA, Molecular Probes) was used to label cone pedicles.

### Image analyses

To measure the distance of EGFP-KASH2^+^ rod and cone nuclei from the outer edge of the ONL, distances between their respective centroids and the outer edge of the ONL (drawn in the DAPI channel using NIS-Elements quantification tools (Nikon)) were measured. For EGFP-KASH2^+^ rod nuclei, the distribution of measured distances among four equal subdivisions of the ONL (Q1 to Q4) was then compared to a random distribution using a Chi Square test. For cone nuclei, distances measured in LMNA^+/+^, Sun1^+/−^ and Tg(CMV-LacZ/EGFP-KASH2) retinas were used to determine their respective inclusion zones defined as the average distance from the ONL ± 2SD. Distances measured in LMNA^−/−^, Sun1^−/−^ and Tg(^HRGP*flox*^CMV/EGFP-KASH2) littermates retinas were then used to determine whether a given cone centroid localized either within or outside the inclusion zone. To compare the intensity of apical vs. basal EGFP-KASH2^+^ cone nuclei within Tg(^HRGP*flox*^CMV/EGFP-KASH2) retinas, sum intensities of EGFP-KASH2^+^ nuclei was quantified, and normalized to the total area of the nucleus. Background signal, averaged from five cone nuclei-free areas per retina field, was substracted from average normalized intensities. Mean background-corrected values were then calculated for ectopic nuclei and nuclei residing within the inclusion zone. Maximum Feret diameters of apical vs. basal EGFP-KASH2+ cone nuclei were measured in Tg(^HRGP*flox*^CMV/EGFP-KASH2) retinas by applying the appropriate macros of NIS-elements on nuclei whose perimeters were drawn over EGFP-KASH2^+^ nuclear rims. To compare the size of cone populations between Tg(^HRGP*flox*^CMV/EGFP-KASH2) and Tg(CMV-LacZ/EGFP-KASH2) littermates, the number of cone outer segments stained with anti-cone arrestin was counted in two retinas from each genotype. Sections lengths were measured by tracing the apical edge of DAPI stained ONL. Average cone numbers per 100 μm of retinal sections were then estimated. To quantify PNA signal intensities underneath EGFP-KASH2^+^ nuclei, maximum intensity projections of Z-stacks were acquired from Tg(^HRGP*flox*^CMV/EGFP-KASH2) retinas stained with PNA. To compare PNA signals intensities associated either to EGFP-KASH2^+^ cone nuclei mislocalizing within the OPL or to cone pedicles from regions devoid of EGFP-KASH2^+^ nuclei, background-corrected mean intensities were measured using appropriate macros (NIS-Element, Nikon). 3D renditions of corresponding Z-stacks were used to unequivocally associate a PNA signal underneath a given EGFP-KASH2^+^ cone nucleus. 3D rendering of CAR and PNA signals within Tg(^HRGP*flox*^CMV/EGFP-KASH2) retinas (Movies S1 and S2) were built using the filmmaker macro within NIS-Element.

## Results

### Development of a new mouse model of inducible LINC complex disruption

We previously showed that, in cultured mammalian cells, the forced expression of the KASH domain of Nesprin1, 2 or 3 fused to EGFP competes with endogenous SUN/KASH interactions at the NE and invariably leads to the displacement of endogenous Nesprins from the NE to the ER [15], a phenomenon we call disruption of LINC complexes. These data therefore suggested that conditional transgenic expression of KASH domains could achieve the spatiotemporal disruption of LINC complexes in vivo. Hence, we raised transgenic mice harboring a genetic cassette (Fig. 1B) consisting in the KASH domain of mouse Nesprin2 fused to EGFP (EGFP-KASH2) cloned downstream from a LoxP-flanked open reading frame encoding β-galactosidase fused to a V5 epitope (LacZ/V5) [27]. In these conditions, Cre recombinase-mediated somatic excision of the LacZ/V5 open reading frame should induce the expression of EGFP-KASH2 from the CMV promoter in a tissue and/or cell-specific manner. LacZ/V5 was expressed in a mosaic pattern within the retina of one Tg(CMV-LacZ/EGFP-KASH2) founder (Fig. 1B, left panel). Vertical sections further indicated that transgenic cells strictly originated from the ONL (Fig. 1B, right panel). Co-staining of vertical slices with V5 and cone arrestin reported transgenic expression mostly in rods and only in a few cones (Fig. 1C and Fig. S1).

### Disruption of LINC complexes in photoreceptor cells

**Figure 2:**
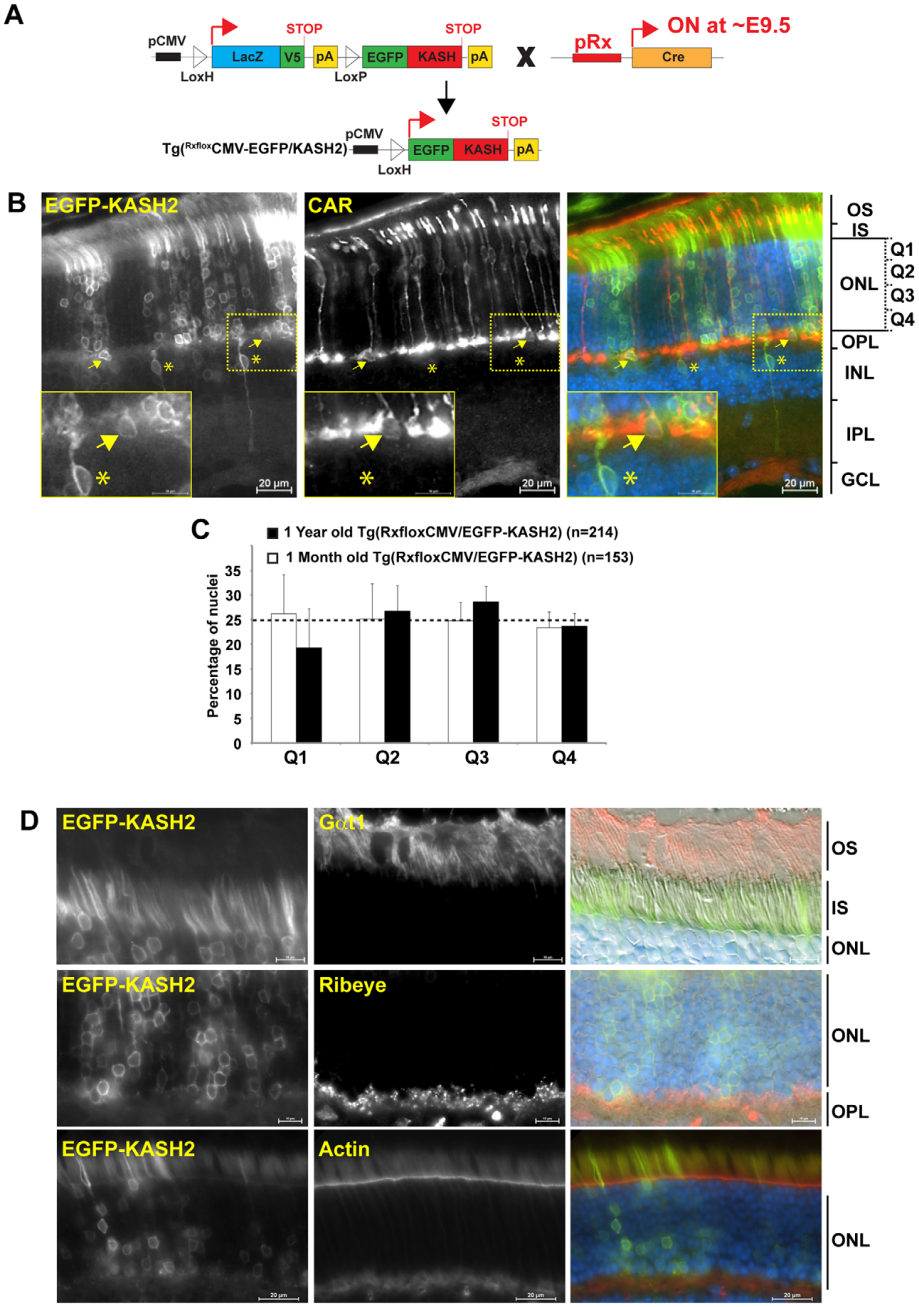
A) Genetic strategy used to derive Tg (*^Rxflox^*CMV-EGFP-KASH2) mice (see text for details). B) Vertical sections of 5 month-old Tg (*^Rx^* ^flox^CMV-EGFP-KASH2) retinas showing the localization of EGFP-KASH2^+^ rims around rod photoreceptor nuclei. Arrows point to CAR^+^/EGFP-KASH2^+^ cone photoreceptors whose nuclei are mispositioned at the basal side of the ONL. Asterisks denote non-photoreceptor cells expressing EGFP-KASH2. OS: outer segment; IS: inner segment; OPL, IPL: outer and inner plexiform layer, respectively. Scale bars: 20 μm and 10 μm (inset). C) EGFP-KASH2 expression in rods does not induce nuclear mislocalization. Distribution of EGFP-KASH2^+^ rod nuclei populations among 4 equal subdivisions of the ONL (Q1, Q2, Q3 and Q4, fig. 2B) from one-month and one-year-old Tg(*^Rx^* ^flox^CMV-EGFP-KASH2) retinas. Error bars represent ±SD from three random observation fields for each genotype. Distributions of EGFP-KASH2+ rod nuclei were not significantly different than a random distribution (Chi Square, p>0.05). D) EGFP-KASH2 overexpression does not affect rod overall morphology and outer limiting membrane (OLM) integrity. One year-old Tg(*^Rxflox^*CMV-EGFP-KASH2) retinas were immunolabeled with Gαt1 (rod outer segment), Ribeye (photoreceptors synaptic ribbons) and Texas-Red Phalloidin (actin component of OLM).

To examine the full expression pattern of EGFP-KASH2 in mouse retina, Tg(CMV-LacZ/EGFP-KASH2) mice were initially bred to Tg(Rx-Cre) mice that initiates the expression of Cre recombinase at ∼E9.5 in most cells of the optic field (Fig. 2A) [30]. As shown in figure 2B, vertical slices from floxed retinas (called Tg(^Rx*flox*^CMV-EGFP-KASH2) hereafter) displayed EGFP-KASH2^+^ rim-like patterns reminiscent of perinuclear EGFP-KASH2^+^ rims observed in transfected mammalian cells [15]. EGFP-KASH2 was mostly expressed in rods that account for 97% of the neuronal population within the ONL of mouse retina [2]. To examine whether the expression of EGFP-KASH2 in rods altered the positioning of their nuclei across the ONL, the distribution of EGFP-KASH2^+^ rod nuclei among 4 equal ONL bins (Q1 to Q4, Fig. 2B) was measured in one month- and one year-old Tg(^Rx*flox*^CMV-EGFP-KASH2) retinas (Fig. 2C). The fraction of EGFP-KASH2^+^ rod nuclei within each bin was not significantly different than a random distribution predicting a quarter of the total EGFP-KASH2^+^ rod nuclei population within each bin. These results indicated that rod nuclei localization within the ONL is not affected by EGFP-KASH2 expression. Examination of EGFP-KASH2^+^ rods in one year-old Tg(^Rx*flox*^CMV-EGFP-KASH2) retinas with rod outer segments (Fig. 2D, upper panel) and photoreceptor synaptic terminals (Fig. 2D, middle panel) markers did not reveal any obvious alteration of the overall architecture of EGFP-KASH2^+^ rod photoreceptors. Furthermore, the outer limiting membrane (OLM), which separates inner and outer segments from photoreceptor somas, was structurally intact (Fig. 2D, lower panel). Taken together, these results indicated that EGFP-KASH2 expression was efficiently induced upon Cre-mediated recombination in rod photoreceptors and that, if expressed in rods (see below), LINC complexes do neither play a role in nuclear positioning nor in the overall structural integrity of rod photoreceptors.

While transgenic expression of LacZ/V5 was restricted to the ONL of Tg(CMV-floxLacZ-EGFP/KASH2) mice (Fig. 1B, C), a significant number of EGFP-KASH2^+^ nuclei were observed in the INL of Tg(^Rx*flox*^CMV-EGFP-KASH2) retinas (Fig. 2B, asterisks). Subpopulations of these nuclei corresponded to Müller and rod bipolar cells (data not shown). While we did not further investigate this observation, we currently hypothesize that INL neurons expressing EGFP-KASH2 are either not detected through Xgal and V5 staining within Tg(CMV-floxLacZ-EGFP/KASH2) retinas (Fig. 1B,C) or originate from a modification of cell fate decision of the few retinal cell precursors (RPC) expressing EGFP-KASH2 in Tg(^Rx*flox*^CMV-EGFP-KASH2) embryonic retinas (Fig. S2). Consistent with that notion, alteration of LINC complexes within Zebrafish retinal progenitor cells modifies cell fate decision [32].

### LINC complexes mediate the positioning of cone photoreceptors nuclei on the apical side of the ONL

**Figure 3:**
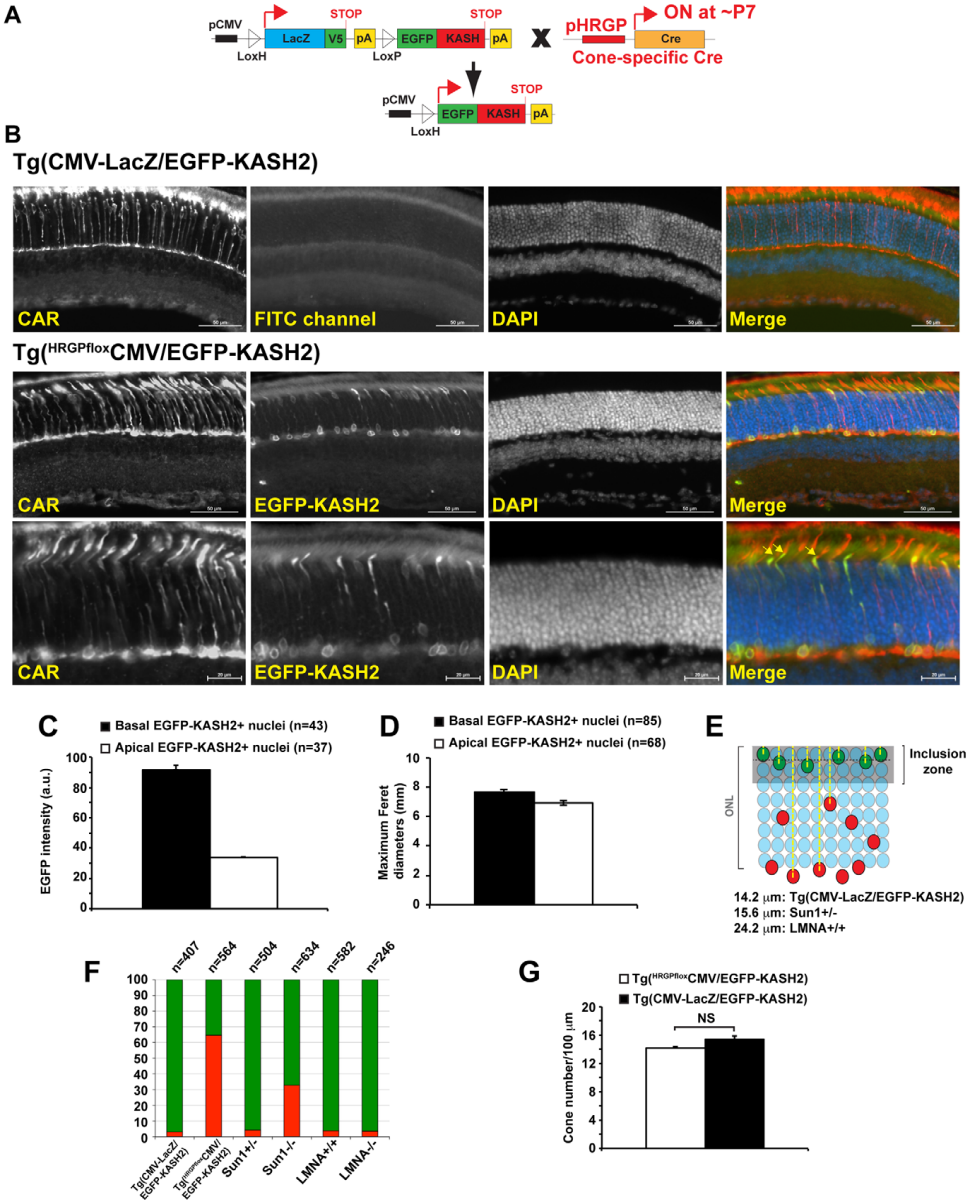
LINC complexes mediate the positioning of cone photoreceptor nuclei. A) Genetic strategy used to derive Tg(*^HRGP^* ^flox^CMV-EGFP-KASH2) mice expressing EGFP-KASH2 specifically in cone photoreceptors. B) CAR immunostaining of P26 Tg(CMV-LacZ/EGFP-KASH2) and Tg(*^HRGP^* ^flox^CMV-EGFP-KASH2) littermates retinas. Lower panel: Zoomed view of the basal side of the ONL showing CAR^+^/EGFP-KASH2^+^ nuclei in the outer plexiform layer. Yellow arrows in merged image point to OS atop IS of cone nuclei expressing high levels of EGFP-KASH2. Scale bars: 50 μm and 20 μm (lower panel). C) Basalmost EGFP-KASH2^+^ cone nuclei express a significantly higher level of EGFP-KASH2 recombinant protein in comparison to their apical counterparts (p<0.001, Student's t-test). Error bars represent ±SEM from measurement of EGFP intensities of basal and apical nuclei from three random fields within ONL two Tg(*^HRGP^* ^flox^CMV-EGFP-KASH2) littermate retinas. D) Mispositioned EGFP-KASH2^+^ nuclei are significantly less elongated. Maximal Feret diameters were significantly smaller in basal (n = 85) vs. apical (n = 68) EGFP-KASH2+ nuclei (p<0.01, Student's t-test). Error bars represent ±SEM from measurements of two random fields within two Tg(*^HRGP^* ^flox^CMV-EGFP-KASH2) littermate retinas. E) Depiction and measurement of inclusion zones for the indicated genotypes (see text for details). Red nuclei are ectopic (centroids outside the inclusion zone) while green nuclei are correctly positioned (centroids within the inclusion zone). F) Percentages of ectopic (red) and of correctly positioned (green) nuclei of populations of n cone nuclei of the indicated genotypes. G) The size of cone populations estimated by the number of cone outer segments labeled with CAR in 4 month-old Tg(CMV-LacZ/EGFP-KASH2) and Tg(*^HRGP^* ^flox^CMV-EGFP-KASH2) littermates retinas are not significantly different (p>0.05, Student's t-test). Error bars represent ±SEM from the counting of 5 random fields within littermate retinas of each genotype.

Within Tg(^Rx*flox*^CMV-EGFP-KASH2) retinas, we observed a few EGFP-KASH2^+^ nuclei that were positive for cone arrestin (CAR), a cone-specific marker. By contrast to non-transgenic cone nuclei, these nuclei mislocalized on the basal side of the ONL (Fig. 2B, arrows) suggesting that the expression of EGFP-KASH2 induces the mislocalization of cone nuclei. To further examine this phenotype and exclude the contribution of cell nonautonomous effects, Tg(CMV-LacZ/EGFP-KASH2) were bred to Tg(HRGP-Cre) mice [29] that initiate the cone-specific expression of Cre recombinase at ∼P7 (Fig. 3A). As expected, Tg(CMV-LacZ-EGFP/KASH2) retinas did not show any expression of EGFP-KASH2 and cone nuclei appropriately localized on the apical side of the ONL (Fig. 3B, upper panels). By contrast, retinas from Tg(^HRGP*flox*^CMV/EGFP-KASH2) littermates displayed EGFP-KASH2^+^ nuclear rims within the ONL (Fig. 3B, middle panel). The population of EGFP-KASH2^+^ nuclei in the ONL of Tg(^HRGP*flox*^CMV/EGFP-KASH2) retinas was much larger than the small population of transgenic cones we detected in Tg(CMV-LacZ/EGFP-KASH2) retinas using either Xgal staining or V5 immunostaining (Fig. 1B, C). Hence, we confirmed that all EGFP-KASH2^+^ nuclei expressed either CAR (Fig. 3B, lower panel) or cone opsin (data not shown). These results therefore emphasize that Xgal staining and V5 immunostaining of Tg(CMV-LacZ/EGFP-KASH2) retinas did not reliably reflect the actual size of transgenic cone populations.

Two distinct populations of EGFP-KASH2^+^ cone nuclei were observed in Tg(^HRGP*flox*^CMV/EGFP-KASH2) retinas. One population, which expressed low levels of EGFP-KASH2 localized normally on the apical side of the ONL while the other, which expressed higher levels of EGFP-KASH2, localized ectopically on the basal side of the ONL (Fig. 3B, middle and lower panels). Many basalmost cone nuclei were actually found within the outer plexiform layer (OPL). The fact that these nuclei expressed both EGFP-KASH2 and cone arrestin (Fig. 3B, lower panels) confirmed that they did not correspond to nuclei from INL cells. As shown in Figure 3C, basal cone nuclei expressed on average 3 times more EGFP-KASH2 than their apical counterparts indicating that a certain threshold of EGFP-KASH2 expression is required to induce the mislocalization of cone nuclei. Ectopic EGFP-KASH2^+^ cone nuclei were significantly less elongated as indicated by lower values of their maximum Feret diameters that correspond to the longest distance between any two points of a given nucleus (Fig. 3D). Furthermore, while not significant at the population level, we often observed basalmost EGFP-KASH2+ nuclei whose longer axis was quasi perpendicular to the longest axis of their apical counterparts (Fig. 3B, middle panel). Taken together, and in agreement with the ectopic localization of EGFP-KASH2^+^ cone nuclei observed in Tg(^Rx*flox*^CMV-EGFP-KASH2) retinas (Fig. 2B), these results demonstrate that EGFP-KASH2 overexpression targeted to cone photoreceptors induces the basal mislocalization of their nuclei in a cell autonomous manner.

To quantify the extent of cone nuclei mispositioning, we defined an “inclusion zone”, measured within wild-type littermates of a given genotype, that corresponds to the average distance ±2SD between cone nuclei centroids and the outer edge of the ONL (Fig. 3E). Any cone nucleus was considered “ectopic” when the position of its centroid fell outside the inclusion zone. In Tg(CMV-LacZ/EGFP-KASH2) retinas, cone nuclei centroids were located at 7+/−3.6 μm from the outer edge of the ONL, thereby delineating an inclusion zone of 14.2 μm. Using these criteria, 3.1% of CAR^+^ cone nuclei mislocalized outside this inclusion zone in Tg(CMV-LacZ/EGFP-KASH2) retinas (Figs. 3F, S4A). By contrast, more than 60% of EGFP-KASH2^+^ cone nuclei fell outside the inclusion zone in Tg(^HRGP*flox*^CMV/EGFP-KASH2) retinas (Figs. 3F, S4A).

Outer segments of EGFP-KASH2^+^ cones did not present any obvious structural alteration. Indeed, anti-CAR antibody homogenously labeled outer segments atop inner segments expressing high levels of EGFP-KASH2 (Fig. 3B, arrows in merged lower panel). Immunolabeling of Tg(^HRGP*flox*^CMV/EGFP-KASH2) retinal flat mounts with CAR further confirmed that the structural integrity of cone OS expressing EGFP-KASH2 was preserved (Fig. S3). Furthermore, the number of cone outer segments was not significantly different in 4 month-old Tg(^HRGP*flox*^CMV/EGFP-KASH2) by comparison to Tg(CMV-LacZ/EGFP-KASH2) littermates retinas indicating that cone degeneration is not at play (Fig. 3G). From these observations, we conclude that EGFP-KASH2 expression drastically alters the positioning of cone nuclei but affects neither the structural organization of cones photosensitive region nor cone photoreceptors survival.

### Disruption of LINC complexes prevents cone precursor nuclei migration towards the apical side of the developing ONL

**Figure 4:**
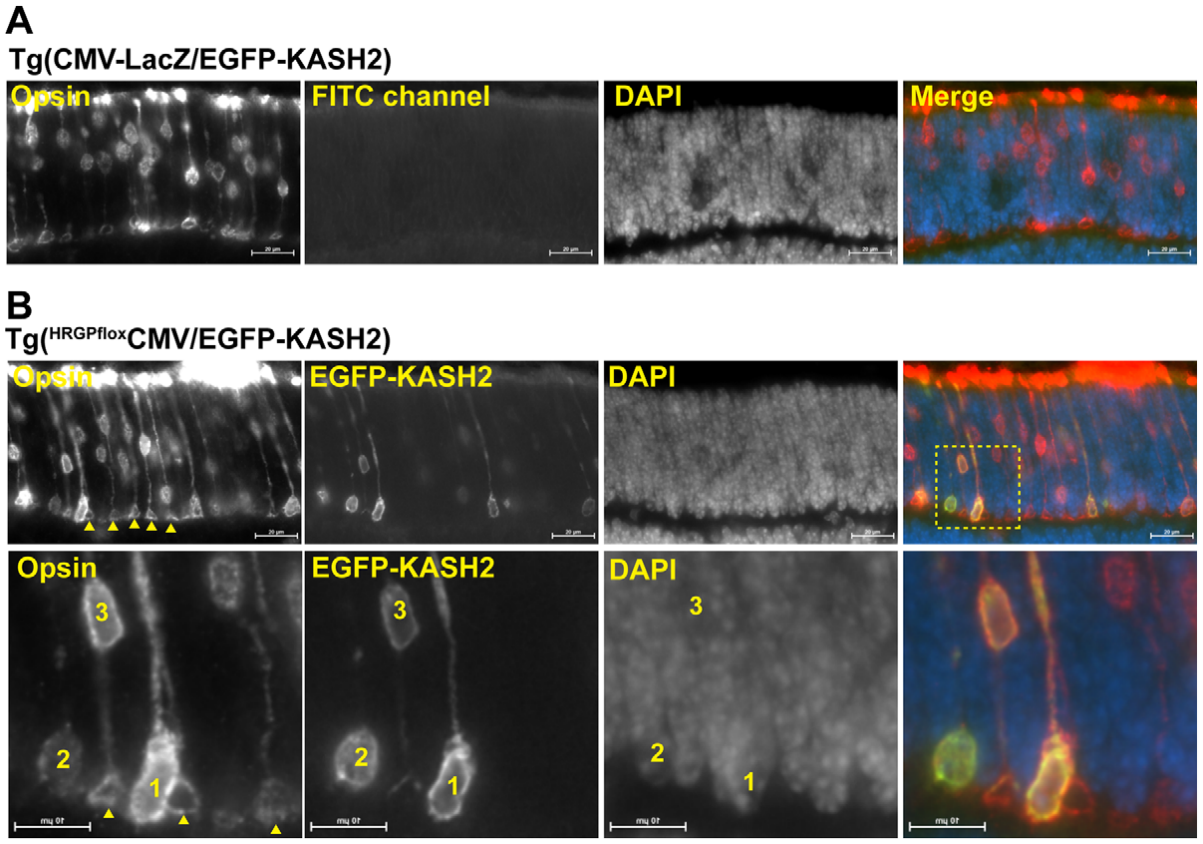
EGFP-KASH2^+^ cone precursor nuclei fail to migrate towards the apical surface of the developing ONL. A) Cone opsin staining of P8 Tg(CMV-LacZ/EGFP-KASH2) retinas. Note the scattering of wild-type cone nuclei within the apical two thirds of the developing ONL. B) Same experiment on P8 Tg(*^HRGP^* ^flox^CMV-EGFP-KASH2) littermate retinas. Note the basalmost mislocalization of cone nuclei expressing high levels of EGFP-KASH2. Arrowheads point to pyramid-shaped pedicles. Inset (lower panels): pyramid-shaped pedicles are present beneath wild type or EGFP-KASH2^+^ nuclei that are still confined within the ONL (nucleus 3) but not beneath EGFP-KASH2^+^ cones whose nuclei occupy basalmost locations (nuclei 1 and 2). Scale bars: 20 μm (upper panel) and 10 μm (lower panel).

Between postnatal day 4 and 11, cone precursor nuclei are scattered across the developing ONL before migrating towards apical positions at P12 [33], [34]. Hence, we examined whether the ectopic localization of EGFP-KASH2^+^ cone nuclei observed in adult retina originated either from the inability of EGFP-KASH2+ cone precursor nuclei to migrate towards the apical side of the ONL during postnatal development or from a post-migratory loss of anchorage of differentiated cone nuclei at the apical side of the ONL. For that purpose, the positioning of EGFP-KASH2^+^ nuclei was examined in P8 retina, i.e., 1 day after the onset of Cre expression in Tg(^HRGP*flox*^CMV/EGFP-KASH2) retinas. Within P8 Tg(CMV-LacZ/EGFP-KASH2) retinas, cone precursors were indeed scattered across the apical two third of the developing ONL (Fig. 4A). By contrast, in P8 Tg(^HRGP*flox*^CMV/EGFP-KASH2) retinas, cone precursor expressing high levels of EGFP-KASH2 already mislocalized at basalmost locations of the developing ONL (Fig. 4B). These results suggest a model whereby the mispositioning of EGFP-KASH2^+^ nuclei originates from the inability of EGFP-KASH2+ nuclei to migrate towards the apical side of the forming ONL during postnatal retinal development.

### Cone nuclei mispositioning affect pedicle architecture

**Figure 5:**
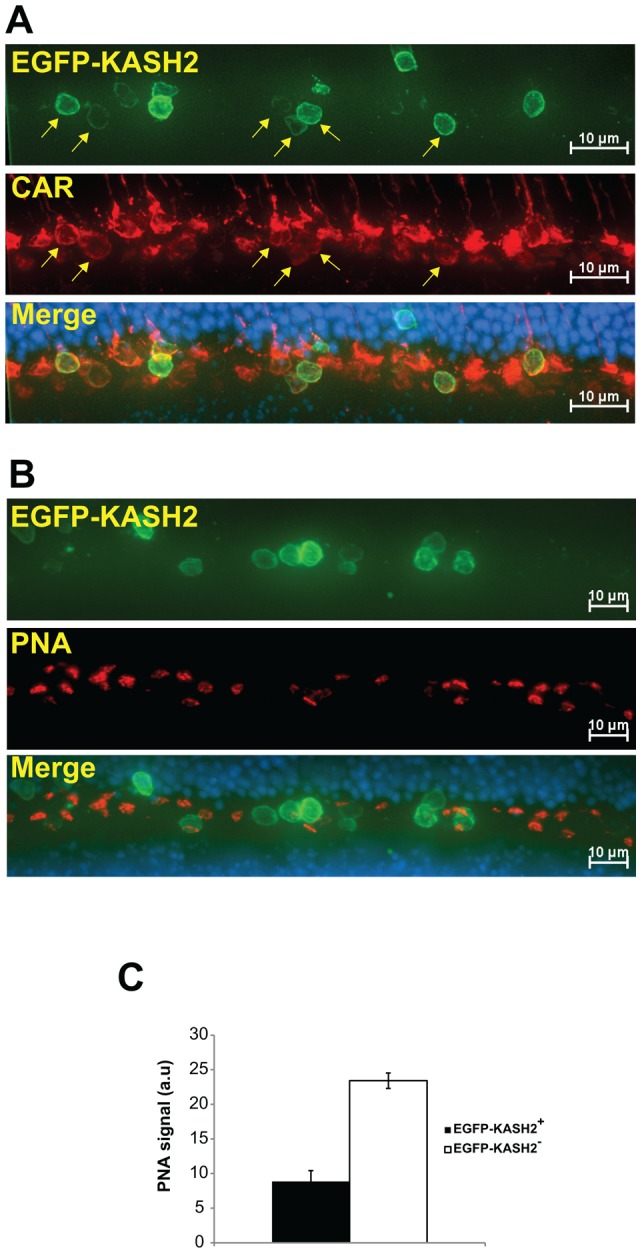
Basalmost localization of EGFP-KASH2+ cone nuclei alters cone terminals morphology in adult Tg(*^HRGP^* ^**flox**^CMV-EGFP-KASH2) retina. A) Maximum intensity projection of a Z-stack series acquired from Tg(*^HRGP^* ^flox^CMV-EGFP-KASH2) retina stained with either anti-CAR (A) or Alexa594-PNA (B). Arrows in A) denote the staining pattern of CAR underneath EGFP-KASH2^+^ cones nuclei located within the OPL. As shown in B), these nuclei also displayed weaker or no basal PNA signal. Sale bars: 10 μm. C) PNA signal underneath EGFP-KASH2^+^ cone nuclei located within the OPL (EGFP-KASH2^+^) is significantly weaker (p<0.01) by comparison to PNA signals measured from regions devoid of EGFP-KASH2^+^ nuclei (EGFP-KASH2^−^).

In P8 retinas, opsin antibodies clearly labeled pyramid-shaped cone pedicles (arrows, Fig. 4B) beneath EGFP-KASH2^+^ cone nuclei that did not localize at the basal edge of the developing ONL (Fig. 4B, nucleus 3). By contrast, cones with basalmost EGFP-KASH2^+^ nuclei did not display such structures (Fig. 4B, nuclei 1&2). These results suggested that basalmost EGFP-KASH2^+^ nuclei interfere with cone terminals architecture. Hence, we examined the structural organization of cone terminals in adult Tg(^HRGP*flox*^CMV/EGFP-KASH2) retinas. While cone arrestin (CAR) strongly labeled thick pedicles in cones devoid of basal most nuclei, we observed a much weaker CAR signal that “wrapped” EGFP-KASH2^+^ cone nuclei mislocalizing within the OPL (Fig. 5A and Movie S1). Alexa594-conjugated Peanut Agglutinin (PNS) was then used to examine the active zone of cone pedicles. As shown in figures 5B and 5C, PNA fluorescence was either significantly weaker or absent beneath EGFP-KASH2^+^ cone nuclei that mislocalized within the OPL (see also Movie S2). Taken together, these results indicate that mislocalization of EGFP-KASH2^+^ cone nuclei within the OPL interferes with the overall architecture of cone pedicles.

### Sun1, but not A-type lamins, participates in the positioning of cone photoreceptor nuclei

**Figure 6:**
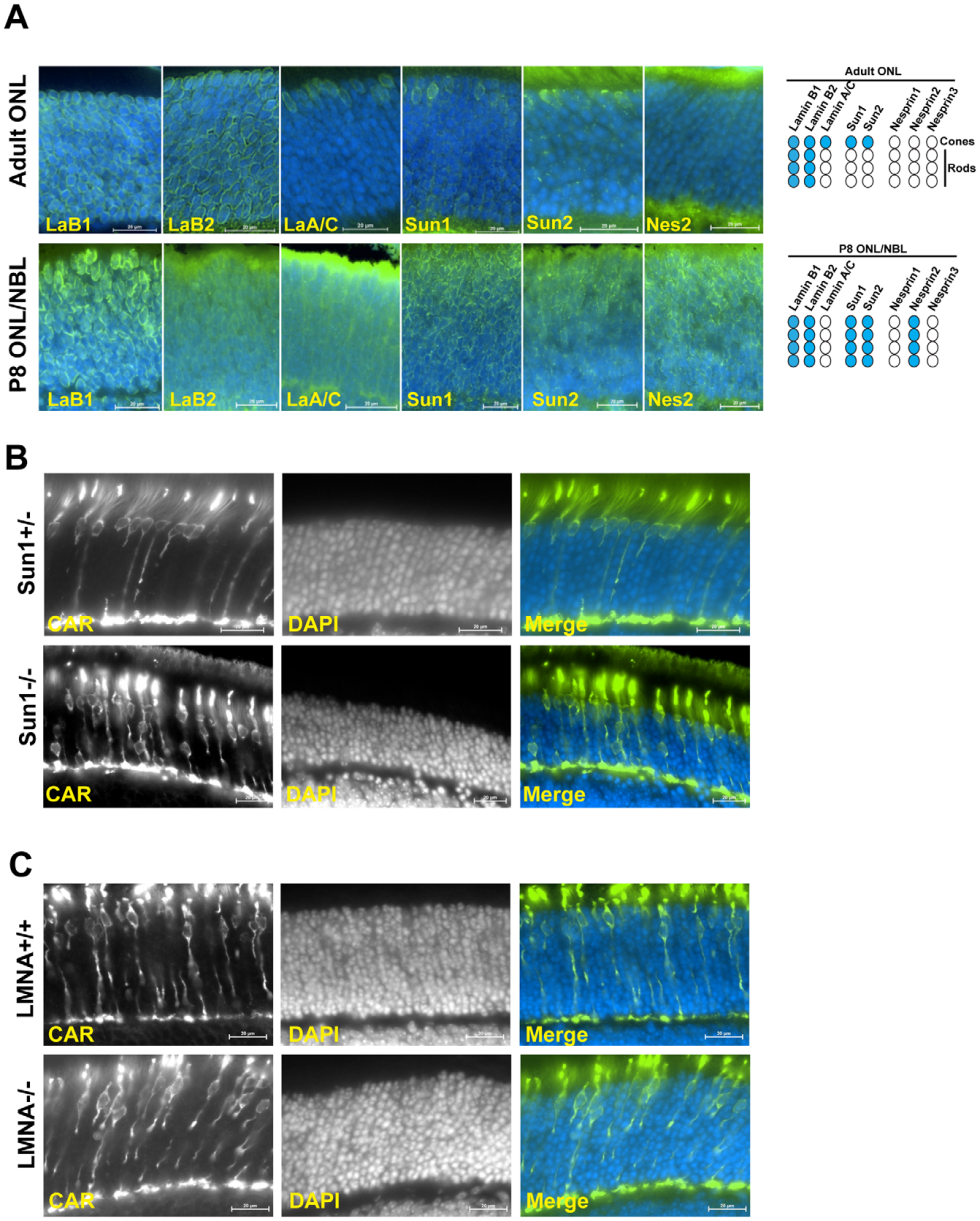
Sun1, but not A-type lamins, participates in the positioning of cone photoreceptor nuclei. A) Immunolocalization of A- and B-type lamins, Sun1, Sun2 and Nesprin2 within the mature ONL of adult retinas (top) or the developing ONL of P8 retinas (bottom). Cartoons: summary of immunolocalization experiments (blue: positive, white: negative). Scale bars: 20 μm. B, C) Immunolocalization of cone nuclei within the ONL of P32 Sun1^−/−^ (B) and P21 LMNA^−/−^ (C) retinas in comparison to their respective wild-type littermates. See Figure 3F and S4 for quantification of cone nuclei positioning in these genotypes. Scale bars: 20 μm.

The expression pattern of lamins and LINC complex components was examined within the ONL of wild-type adult retinas (Fig. 6A, upper panel). B-type lamins were ubiquitously expressed in all nuclei of the ONL. However, we were unable to detect any significant expression of Nesprin 1, 2 or 3 within the ONL of adult retinas even though these antisera detected nuclear rims within other retinal neurons (Fig. 6A and data not shown). Because A-type lamins and Sun1 were specifically detected around cone nuclei (Fig. 6A, top panels), we examined the positioning of cone nuclei within Sun1 and LMNA KO mice retinas.

In Sun1^+/−^ adult retinas, cone nuclei centroids were located at an average distance of 7+/−4.3 μm from the outer edge of the ONL (Fig. 6B) thereby delineating an inclusion zone of 15.6 μm (Fig. 3E). Within Sun1^−/−^ retinas, 32.8% of cone nuclei localized outside the inclusion zone (Fig. 3F, S4B) confirming recent observations that Sun1 contributes to the positioning of cone nuclei [22]. However, it is important to note that this percentage was significantly lower than the percentage of ectopic KASH2^+^ cone nuclei measured within Tg(^HRGP*flox*^CMV/EGFP-KASH2) (Figs. 3F, S4). In addition, very few cone nuclei mislocalized within the OPL of Sun1^−/−^ retinas. Together, these results suggest that, besides Sun1, another SUN domain-containing protein, which is also saturated by EGFP-KASH2 expression in cones, may act redundantly with Sun1 to mediate cone nuclei positioning. This protein most likely corresponds to Sun2 that, along with Sun1, was detected as nuclear rims both within the developing ONL of P8 retinas and specifically in cones within adult retinas (Fig. 6A).

LMNA^−/−^ retinas did not display any cone nuclei mispositioning phenotype (Figs. 6C, 3F and S4C) indicating that A-type lamins are dispensable cone nuclei positioning. In support of that result, A-type lamins could not be detected within P8 ONL while they were specifically expressed in adult cones. These data indicate that A-type lamins do not mediate cone nuclei positioning. Of note is that because LMNA^−/−^ mice die within 3 to 4 weeks, P21 retinas were used to measure cone nuclei positioning for that genotype, i.e., about a week earlier than all other genotypes analyzed in this study. Interestingly, the average cone nuclei centroids distance from the outer edge of the ONL of these retinas was significantly larger in P21 LMNA^+/+^ retinas by comparison to P32 Sun1^+/−^ and P26 Tg(CMV-LacZ/EGFP-KASH2) retinas (Fig. 3E, S4). These results indicate that cone nuclei migration towards the apical side of the ONL is therefore not yet fully achieved by P21.

## Discussion

In this work, we developed a new mouse model to induce the disruption of LINC complexes in a cell type-specific manner *in vivo*. This approach is based on the dominant negative effect that exogenous recombinant KASH domains exert on evolutionary-conserved endogenous SUN/KASH interactions [14], [15], [19], [35], [36]. Importantly, by comparison with current mouse models of germline mutation of individual Sun proteins and Nesprins, this model overcomes the issue of redundancy associated with the multiplicity of genes encoding SUN and KASH domain-containing proteins in mammals, circumvents potential cell non-autonomous effects that complicate phenotypical interpretation and bypasses perinatal lethality. Despite these significant advantages, our approach is not without drawbacks. First, because EGFP-KASH2 disrupts endogenous SUN/KASH interactions as a whole, our approach forgoes the direct identification of Sun protein(s) and Nesprin(s) involved in a given nuclear mislocalization phenotype. To that respect, ongoing studies are aimed at the direct identification of Nesprin protein(s) expressed in cone and rod photoreceptors. Second, the restriction of transgenic expression to the photoreceptor layer (Fig. 1B) and the mosaic expression pattern of EGFP-KASH2 was manifest and most likely originate from position effects of transgene integration and promoter use. Even though such heterogeneous expression can be advantageous to some respect, alternative transgenic strategies are currently being developed in order to express EGFP-KASH2 in a more ubiquitous manner.

We show that transgenic expression of EGFP-KASH2 severely impairs cone photoreceptors nuclei positioning on the apical side of the ONL in adult Tg(^HRGP*flox*^CMV/EGFP-KASH2) retinas. Importantly, this is the first time that this phenotype is observed in a cell autonomous manner. While we did not observe any nuclear mislocalization phenotype in cones expressing EGFP (data not shown), non-specific effects of EGFP-KASH2 transgenic expression on nuclear positioning cannot formally be excluded. However, our observations parallel the failure of photoreceptor precursor nuclei that either lack Klar, a KASH protein, or Klaroid, a SUN protein, to migrate towards the apical surface of developing Drosophila ommatidia. As a result, these nuclei mislocalize in the optic stalk [37], [38]. In Zebrafish, exogenous expression of the KASH domain of a Nesprin ortholog (Syne2a) also induces photoreceptors nuclei mispositioning [39]. In addition to the lack of evidence that SUN/KASH interactions may take place at sites other than the nuclear envelope, it is likely that the nuclear mislocalization phenotypes we observed are specifically due to disruption of endogenous SUN/KASH interactions. Taken together, these data illustrate the evolutionary-conserved role of SUN/KASH interactions in mediating photoreceptors nuclear positioning.

Our data are also in line with the ectopic localization of cone nuclei recently reported in Sun1 and Nesprin2 KO retina [22]. However, our analyses revealed a milder cone nuclei displacement phenotype in Sun1^−/−^ retinas by comparison to Tg(*^HRGP^* ^flox^CMV-EGFP-KASH2) retinas. Furthermore, relatively few Sun1^−/−^ cone nuclei mislocalized within the OPL. These results strongly suggest that, similarly to the mediation of nuclear positioning during CNS and skeletal muscle development [25], [31], Sun1 and Sun2 act redundantly in cone nuclei positioning. Accordingly, Sun2 is co-expressed along with Sun1 in the ONL of both P8 retinas and adult cones (Fig. 6A).

Significant ONL thinning associated with excessive apoptosis occurs in Sun1^−/−^ and Nesprin2^−/−^ retinas [22]. We did not observe any local ONL thinning within Tg(*^Rx^* ^flox^CMV-EGFP-KASH2) adult retinas (Fig. 2B) and the number of cones was not significantly altered in adult Tg(*^HRGP^* ^flox^CMV-EGFP-KASH2) (Fig. 3G). Our observations therefore suggest the possibility that ONL thinning and excessive apoptosis observed within Sun1^−/−^ and Nesprin2^−/−^ retinas originate from more general developmental defects and/or from dysfunction of other retinal cell types required for photoreceptor homeostasis.

**Figure 7:**
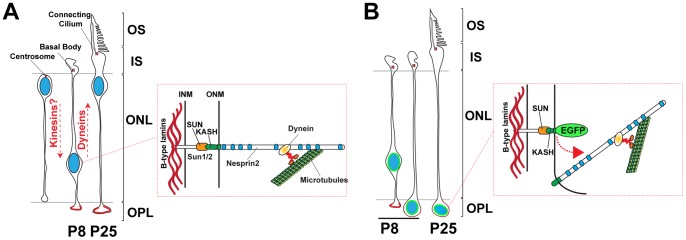
A model for the molecular mechanism underlying the baso-apical migration of cone precursor nuclei. A) Between P4 and P12, cone precursors nuclei initially move towards the basal side of the developing ONL, a movement potentially mediated by microtubules plus-end directed kinesins, before moving back to the apical side. Inset: Depiction of a B-type lamins/Sun1-2/Nesprin2 network of macromolecular complexes that transduce forces generated by dyneins to move cone nuclei precursors back towards the apical side of the developing ONL. B) Disruption of LINC complexes displaces endogenous Nesprin2 (inset) leading to the uncoupling of cone nuclei to dynein. As a result, cone nuclei fail to migrate apically and mislocalize on the inner edge of the ONL. Basalmost localization of these nuclei interferes with the architecture of cone pedicles.

We found that A-type lamins are dispensable for cone nuclei positioning even though A-type lamins interact with the nucleoplasmic domain of Sun proteins in vitro and in immunoprecipitation experiments [9], [10]. The dispensability of A-type lamins in cone nuclei positioning is further suggested by the absence of any obvious CNS defects in newborn LMNA^−/−^ mice that die within ∼4–5 weeks of age from cardiomyopathy and/or muscular dystrophy [28], [40]. By contrast, LMNB1 and LMNB2 KO embryos, similarly to Sun1/2 and Nesprin1/2 DKO embryos [25], display severe neurodevelopmental defects [41], [42] suggesting that B-type lamins act in concert with LINC complexes to position nuclei during neurodevelopment. Accordingly, in Drosophila, mutations in LamDm(0), which encodes a B-type lamin, leads to photoreceptor nuclei mislocalization within the optic stalk of ommatidia [37]. Taken together, these results suggest that forces required for cone precursor nuclei movements are transmitted through LINC complexes and relayed by B-type lamins. Nesprin2 was the only Nesprin we could convincingly detect within the ONL of P8 retinas. Because genetic ablation of Nesprin2 also alters cone nuclei positioning [22], we propose a model whereby macromolecular complexes consisting of B-type lamins/Sun1-Sun2/Nesprin2 underlie cone nuclei migration (Fig. 7A).

KASH proteins interact with kinesins and dyneins in different cellular settings [13], [17], [18], [43]. Furthermore, mutations of dynein subunits impair nuclear mispositioning in Zebrafish retina and Drosophila compound eye [39], [44], [45]. Together, these data suggest that the failure of P8 cone precursor nuclei to migrate apically in developing Tg(*^HRGP^* ^flox^CMV-EGFP-KASH2) retinas as well as nuclear shape abnormalities we observed in basal EGFP-KASH2+ nuclei directly originate from the uncoupling of dynein with the nuclear envelope (Fig. 7B). Interestingly, because EGFP-KASH2^+^ nuclei mislocalize mostly on the basal side of the ONL (Fig. S4), it seems unlikely that LINC complex disruption affects apico-basal migration of cone precursor nuclei. Instead, either a kinesin-based mechanism that would be independent of LINC complexes or a passive apico-basal migration, a phenomenon shown to underlie apico-basal nuclear translocation during interkinetic nuclear migration [46], [47], may be at play (Fig. 7A). As a result, disruption of LINC complexes may prevent to counterbalance forces driving nuclear movement towards the basal side of the developing retina.

By contrast to cones, the positioning of rod photoreceptors nuclei was not affected by EGFP-KASH2 overexpression. In agreement with these results, the expression of Sun1 and Sun2 appears to be progressively downregulated within differentiated rods but maintained in differentiated cones (Fig. 6A). In addition to the lack of any detectable Nesprin 1, 2 and 3 immunoreactivity within differentiated rods, these data suggest that LINC complexes may actually not be expressed at all in rod photoreceptors. Based on these results, we hypothesize that neurons whose nuclei localize at precise laminar positions, such as cone photoreceptors, may be more dependent on LINC complex components for their differentiation and homeostasis. To that respect, Purkinje cells precisely position their nuclei between the molecular and granule layers of cerebellar folds and nonsense mutations of the gene encoding Nesprin1, which is highly expressed in Purkinje cells, have been linked to autosomal recessive ataxia 1 [48], [49]. Our data therefore suggest nuclear mispositioning as a possible molecular etiology of this neurological disorder.

Mislocalized EGFP-KASH2+ nuclei were significantly less elongated and occasionally misoriented. A similar loss of nuclear elongation was observed in basally displaced Zebrafish photoreceptors nuclei [39] and is consistent with the uncoupling of the nucleus with perinuclear cytoskeletal networks reported in cultured cells and metazoans following the expression of KASH domains [20], [50]–[53]. Because such uncoupling induces a disorganization of cytoskeletal networks in cultured cells [35], [51], [53], we anticipated significant architectural abnormalities within EGFP-KASH2^+^ rods and cones. Surprisingly, we found that EGFP-KASH2 expression did not affect the overall organization of rods and cones photosensitive regions. However, the reverse may not be true. Indeed, apical migration of cone precursor nuclei is significantly impaired in mice deficient for Cyclic Nucleotide-Gated Channel 3 (CNGA3^−/−^), an essential component of cone phototransduction, and significantly delayed in *cone photoreceptor function loss 1* (*cpfl1*) mice that carry a spontaneously arising mutation in the cone phosphodiesterase gene and display acute cone degeneration [34], [54]–[56]. Cone populations were not significantly different in Tg(^HRGP*flox*^CMV/EGFP-KASH2) by comparison to Tg(CMV-LacZ/EGFP-KASH2) retinas indicating that cone degeneration is most likely not at play upon EGFP-KASH2+ expression. This result contrasts with the reported degeneration of photoreceptors in Zebrafish retinas following the forced expression of a dominant negative KASH construct [39]. In the latter case, however, cell nonautonomous contributions may adversely affect Zebrafish photoreceptor survival while such contribution can be excluded in Tg(^HRGP*flox*^CMV/EGFP-KASH2) retinas. In mice, whereas Sun1^−/−^ cones appear structurally intact, Nesprin2^−/−^ retina display acute cone OS degeneration [22]. Because cone degeneration was not observed in our study, it seems that genetic inactivation of Nesprin2, by itself, underlies cone OS degeneration. We therefore hypothesize that Nesprin2 genetic inactivation either affects cone OS maintenance in a cell nonautonomous manner or truncates a Nesprin2 KASH-less isoform [57] that may fulfill essential functions in cone OS structural integrity.

Pedicles of EGFP-KASH2^+^ cones appeared normal as long as their displaced nuclei remained within the limits of the ONL. However, EGFP-KASH2^+^ cone nuclei that mislocalized within the OPL appeared to “flatten” pedicles. This suggests that cone terminals architecture is affected not as much as a consequence of EGFP-KASH2 expression but rather as a consequence of acute nuclear mispositioning within the OPL. Whether this observation illustrates a mechanism through which nuclear mispositioning may progressively affect neuronal communication and/or circuitry organization remains to be examined. To that regard, the mispositioning of cone photoreceptor nuclei appears to be a natural phenomenon associated with aging [58]–[60]. Indeed, the mislocalization of human cone photoreceptors nuclei into the OPL occurs at a slow pace early in life but markedly increases by age 30 [58] and even more so in human retina afflicted with age-related macular degeneration [59]. These clinical observations raise the interesting notion of an “aging” LINC complex that would result in a progressive accumulation of mispositioned nuclei. In turn, mislocalized nuclei may potentially interfere with neuronal functions and underlie human retinal diseases as well as other progressive neuronal disorders affecting other regions of the CNS.

## Supporting Information

Figure S1 A) V5 immunostaining of Tg(CMV-LacZ/EGFP-KASH2) retinal flat mount showing the enrichment of transgenic expression on the dorsal side of transgenic retinas. B) Maximum intensity projection of Z-stacks from the apical region of Tg(CMV-LacZ/EGFP-KASH2) retinal flat mounts immunostained with V5 (red) and cone opsin (green). The majority of transgenic photoreceptors correspond to rods whereas only a few cones (labeled with cone opsin) express V5 (arrowheads).
(TIF)

Figure S2 E14 retinas display only a few EGFP-KASH2 + nuclei. E14 retina from Tg(*^Rxflox^*CMV-EGFP-KASH2) embryos were processed for DAPI staining. Note the paucity of EGFP-KASH2 expressing cells at that development time. Scale bar: 50 μm.
(TIF)

Figure S3 EGFP-KASH2 expression in cones does not affect outer segment architecture. Flat mount of a P36 Tg(*^HRGP^* ^flox^CMV-EGFP-KASH2) retina was processed for CAR immunostaining. Shown is a Z-stacks reconstruction of the photoreceptor side showing intact OS atop EGFP-KASH2 + IS of cone photoreceptors (yellow arrow).
(TIF)

Figure S4 Graphical presentation of cone nuclei centroid positions relative the apical side of the ONL for the indicated genotypes. Measurements were obtained from 5 contiguous viewing fields within the central retina from two different mice for all but the LMNA^−/−^ genotype. Vertical error bars represent the standard deviation of the mean ONL thickness across contiguous viewing fields. Note that, as reported by Yu et al [22], the ONL of Sun1^−/−^ retinas was significantly thinner by comparison to their heterozygous counterparts (p<0.05).
(TIF)

Movie S1 3D rendering of Z-stacks acquired from a 15 μm-thick Tg(*^HRGP^* ^flox^CMV-EGFP-KASH2) retina section stained with CAR. Note the paucity and the presentation of CAR signals underneath EGFP-KASH2^+^ nuclei by comparison to pedicles from regions devoid of EGFP-KASH2^+^ nuclei.
(AVI)

Movie S2 3D rendering of Z-stacks acquired from a 15 μm-thick Tg(*^HRGP^* ^flox^CMV-EGFP-KASH2) retina section stained with PNA. Note the paucity or lack of PNA signals underneath EGFP-KASH2^+^ nuclei by comparison to regions devoid of EGFP-KASH2^+^ nuclei. See Fig. 5C for PNA signal quantification.
(AVI)
