# Supplementary figures and images for: LINC Complexes Mediate the Positioning of Cone Photoreceptor Nuclei in Mouse Retina
Source: PLoS One. 2012 Oct 5;7(10):e47180. doi: 10.1371/journal.pone.0047180 (PMC3465324; doi:10.1371/journal.pone.0047180)

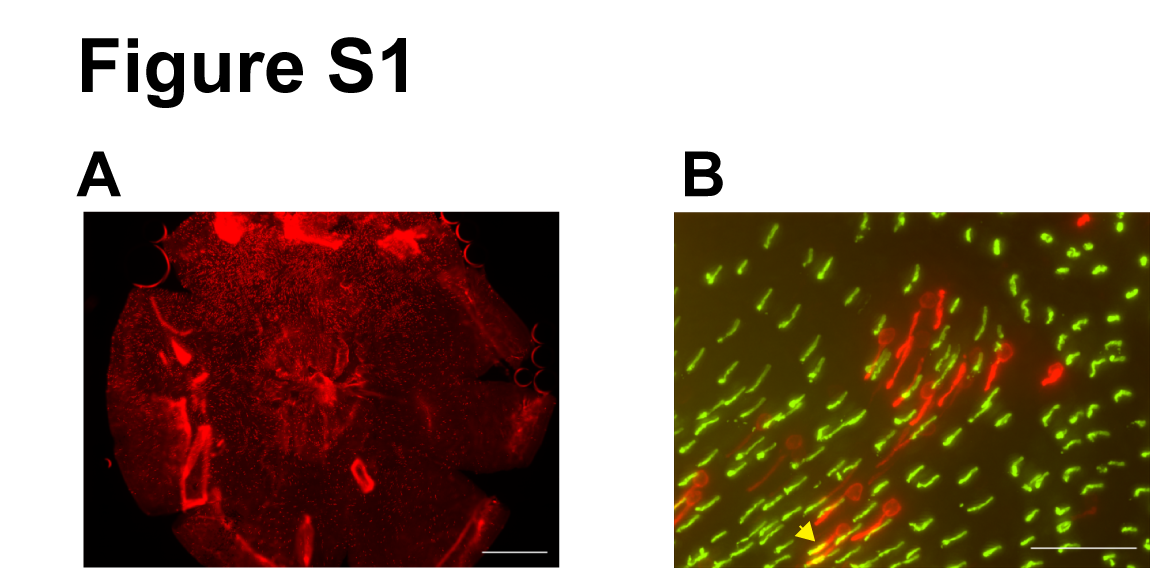

Supplement: Figure S1 — A) V5 immunostaining of Tg(CMV-LacZ/EGFP-KASH2) retinal flat mount showing the enrichment of transgenic expression on the dorsal side of transgenic retinas. B) Maximum intensity projection of Z-stacks from the apical region of Tg(CMV-LacZ/EGFP-KASH2) retinal flat mounts immunostained with V5 (red) and cone opsin (green). The majority of transgenic photoreceptors correspond to rods whereas only a few cones (labeled with cone opsin) express V5 (arrowheads). (TIF) [file pone.0047180.s001.tif]

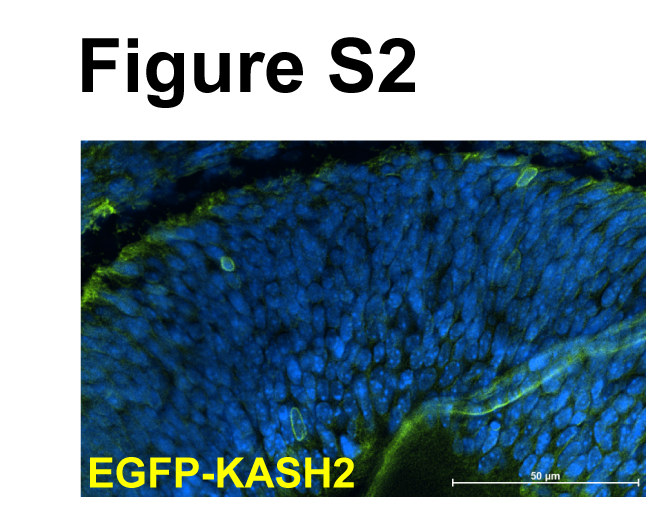

Supplement: Figure S2 — E14 retinas display only a few EGFP-KASH2 + nuclei. E14 retina from Tg(RxfloxCMV-EGFP-KASH2) embryos were processed for DAPI staining. Note the paucity of EGFP-KASH2 expressing cells at that development time. Scale bar: 50 μm. (TIF) [file pone.0047180.s002.tif]

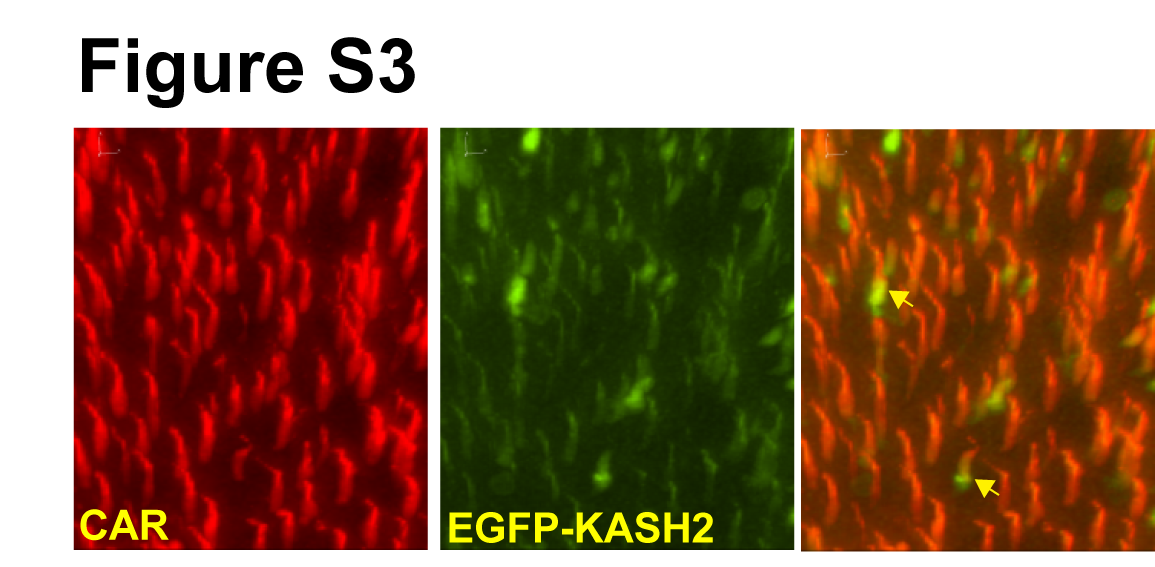

Supplement: Figure S3 — EGFP-KASH2 expression in cones does not affect outer segment architecture. Flat mount of a P36 Tg(HRGP floxCMV-EGFP-KASH2) retina was processed for CAR immunostaining. Shown is a Z-stacks reconstruction of the photoreceptor side showing intact OS atop EGFP-KASH2 + IS of cone photoreceptors (yellow arrow). (TIF) [file pone.0047180.s003.tif]

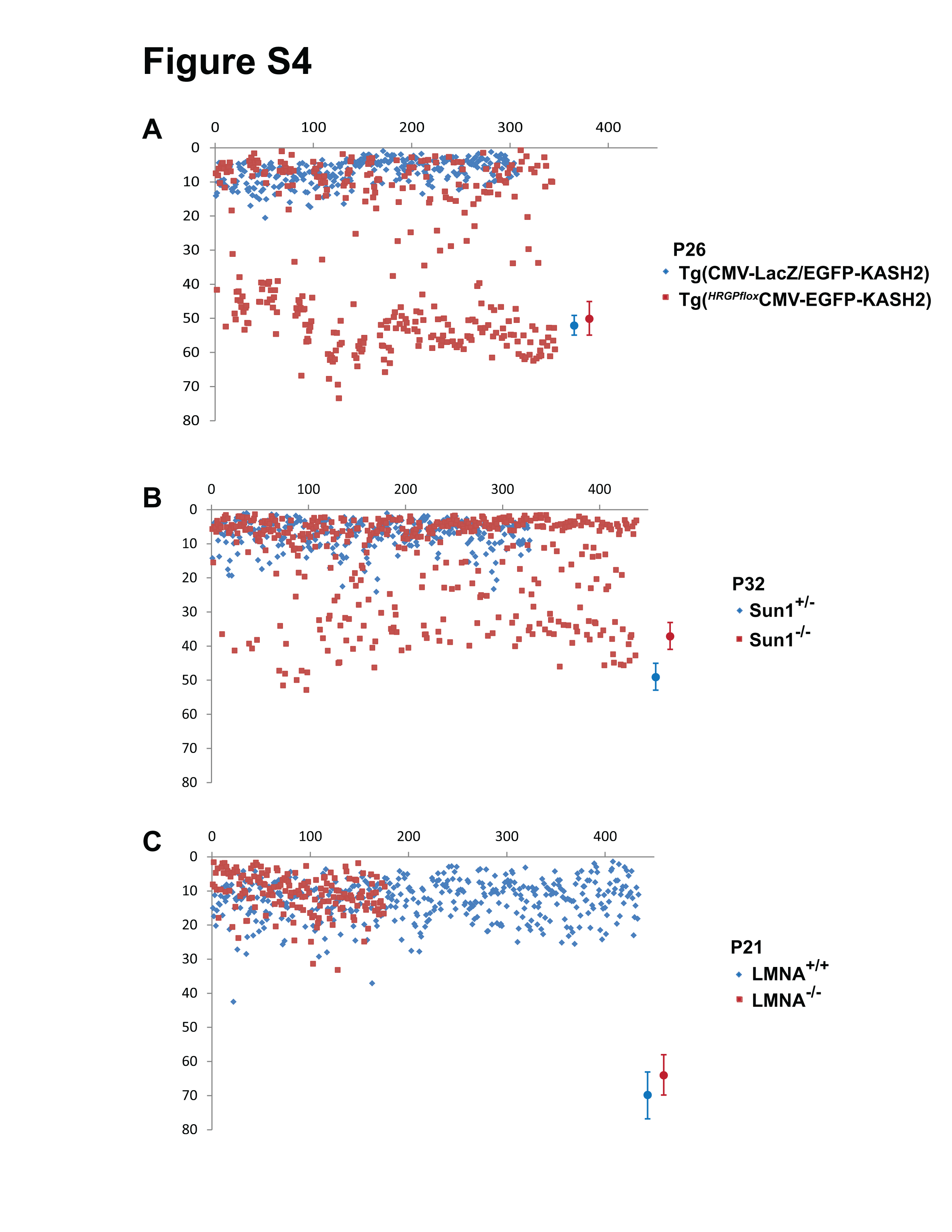

Supplement: Figure S4 — Graphical presentation of cone nuclei centroid positions relative the apical side of the ONL for the indicated genotypes. Measurements were obtained from 5 contiguous viewing fields within the central retina from two different mice for all but the LMNA−/− genotype. Vertical error bars represent the standard deviation of the mean ONL thickness across contiguous viewing fields. Note that, as reported by Yu et al [22], the ONL of Sun1−/− retinas was significantly thinner by comparison to their heterozygous counterparts (p<0.05). (TIF) [file pone.0047180.s004.tif]
